# Crystal structure of 2,3-dimeth­oxy-*meso*-tetra­kis(penta­fluoro­phen­yl)morpholino­chlorin methyl­ene chloride 0.44-solvate

**DOI:** 10.1107/S2056989020009093

**Published:** 2020-07-07

**Authors:** Serena B. S. Churchill, Meenakshi Sharma, Christian Brückner, Matthias Zeller

**Affiliations:** a Department of Chemistry, University of Connecticut, Storrs, Connecticut 06269-3060, USA; b Department of Chemistry, Purdue University, 560 Oval Dr., W. Lafayette, IN 47907-2084, USA

**Keywords:** porphyrinoids, pyrrole-modified porphyrins, morpholino­chlorins, crystal structure

## Abstract

The title morpholino­chlorin, C_46_H_16_F_20_N_4_O_3_, was crystallized from hexa­ne/methyl­ene chloride as its 0.44 methyl­ene chloride solvate, C_46_H_16_F_20_N_4_O_3_·0.44CH_2_Cl_2_. The morpholino­chlorin was synthesized by stepwise oxygen insertion into a porphyrin using a ‘breaking and mending strategy’: NaIO_4_-induced diol cleavage of the corresponding 2,3-di­hydroxy­chlorin with *in situ* methanol-induced, acid-catalyzed intra­molecular ring closure of the inter­mediate secochlorins bis­aldehyde. Formally, one of the pyrrolic building blocks was thus replaced by a 2,3-di­meth­oxy­morpholine moiety. Like other morpholino­chlorins, the macrocycle of the title compound adopts a ruffled conformation, and the modulation of the porphyrinic π-system chromophore induces a red-shift of its optical spectrum compared to its corresponding chlorin analog. Packing in the crystal is governed by inter­actions involving the fluorine atoms of the penta­fluoro­phenyl substituents, dominated by C—H⋯F inter­actions, and augmented by short fluorine⋯fluorine contacts, C—F⋯π inter­actions, and one severely slipped π-stacking inter­action between two penta­fluoro­phenyl rings. The solvate methyl­ene chloride mol­ecule is disordered over two independent positions around an inversion center with occupancies of two × 0.241 (5) and two × 0.199 (4), for a total site occupancy of 88%.

## Abstract 2

The title morpholino­chlorin adopts a ruffled conformation of its porphyrinic π-system chromophore inducing a red-shift of its optical spectrum compared to its chlorin analog.

## Chemical context   

A major aim in contemporary porphyrin chemistry is the generation of NIR (>650 nm) absorbing or fluorescing chromophores for a variety of biomedical and technical applications, such as photodynamic therapy (Dolmans *et al.*, 2003 ▸), solar-energy conversion (Hedley *et al.*, 2017 ▸), or photoacoustic or fluorescence imaging (Gujrati *et al.*, 2017 ▸; Borg & Rochford, 2018 ▸). *Inter alia*, this gave rise to the synthesis of a wide array of porphyrin analogues, including porphyrinoids incorporating non-pyrrolic heterocycles (Brückner *et al.*, 2014 ▸; Lash, 2017 ▸; Chatterjee *et al.*, 2017 ▸).

**Figure 1:**
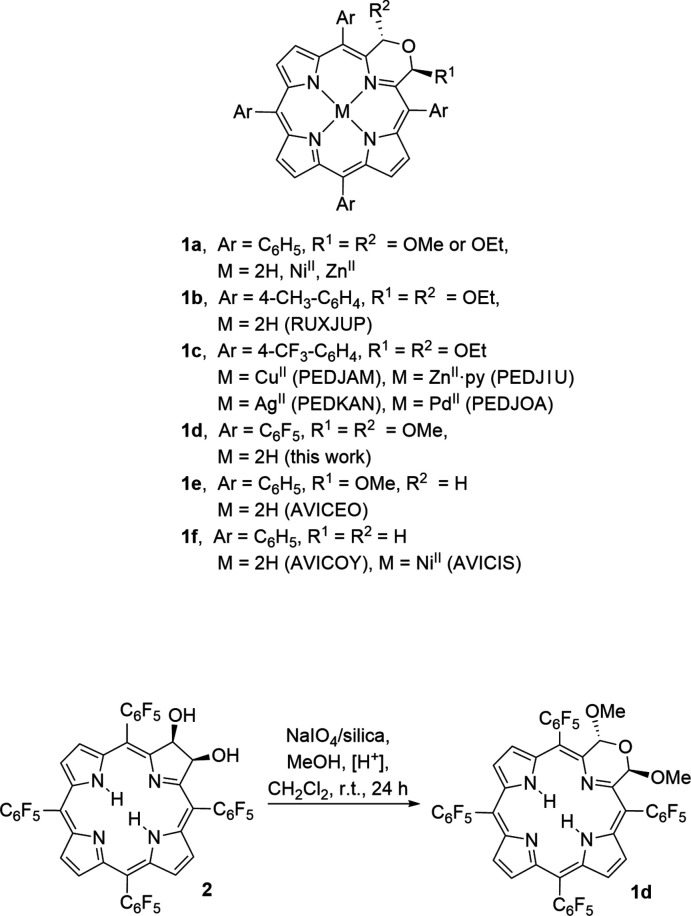
Structures of select morpholino­chlorins

One member of the family of porphyrinoids incorporating non-pyrrolic heterocycles are the morpholino­chlorins (**1**) (Fig. 1 ▸) in which one pyrrolic building block is replaced by a morpholine (Brückner *et al.*, 1998 ▸, 2011 ▸; McCarthy *et al.*, 2003 ▸). This formal replacement is achieved by a stepwise oxygen insertion into a porphyrin using a so-called ‘breaking and mending’ strategy (Brückner, 2016 ▸). As a consequence of the atom insertion, morpholino­chlorins are non-planar (McCarthy *et al.*, 2003 ▸; Brückner *et al.*, 2011 ▸; Sharma *et al.*, 2017 ▸). The twisted (ruffled) conformation of helimeric chirality of the morpholino­chlorins was found to be affected by the size and number of alk­oxy substituents, the presence of covalent links between the morpholine unit and the flanking aryl group, and the presence and type of central metal (Daniell & Brückner, 2004 ▸; Brückner *et al.*, 2011 ▸; Sharma *et al.*, 2017 ▸). Porphyrinoids containing two morpholine moieties are known (Daniell & Brückner, 2004 ▸; Guberman-Pfeffer *et al.*, 2017 ▸), as well as other porphyrinoids containing morpholine building blocks (Lara *et al.*, 2005 ▸; Samankumara *et al.*, 2015 ▸; Akhigbe *et al.*, 2016 ▸). The modulation of the conformation of the porphyrinic π-system also affects their electronic properties; morpholino­chlorins are more red-shifted than a corresponding chlorin (Brückner *et al.*, 2011 ▸; Guberman-Pfeffer *et al.*, 2017 ▸). The influence of the *meso*-substituents on the conformation and electronics of the morpholino­chlorins has not been investigated.

For porphyrinoids at large, the introduction of *meso*-penta­fluoro­phenyl-groups (or fluorine atoms, in general) has long been known to alter their electronic properties (Spellane *et al.*, 1980 ▸; Leroy & Bondon, 2008 ▸; Nardi *et al.*, 2013 ▸); they often become slightly blue-shifted compared to their non-fluorinated analogues and are harder to oxidize. Also, the *meso*-penta­fluoro­phenyl-groups are very convenient handles for the further synthetic manipulation of the porphyrinoids (Costa *et al.*, 2011 ▸; Golf *et al.*, 2015 ▸; Hewage *et al.*, 2015 ▸; Bhupathiraju *et al.*, 2016 ▸). Their effect on the conformation of the mol­ecules, when compared to their hydrogen analogs, has been shown to be frequently minimal (Leroy & Bondon, 2008 ▸).
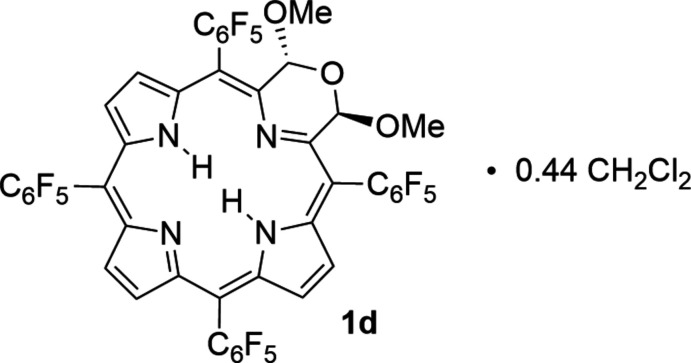


## Structural commentary   

**Figure 2:**
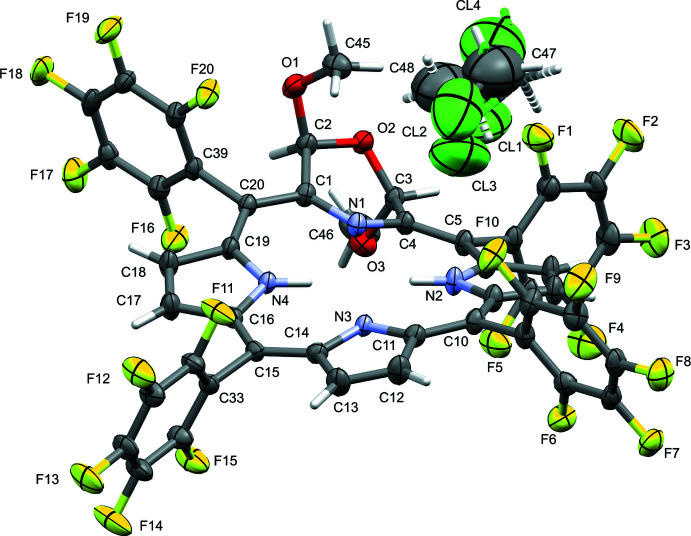
The structure of **1d** with the atom-labeling scheme. Probability ellipsoids are drawn at the 50% level. Symmetry-created atoms are shown in capped-stick mode and are unlabeled. Dashed bonds indicate minor moiety disordered and symmetry-related atoms. Some carbon atom labels are omitted for clarity.

The title compound **1d** was obtained in crystalline form from hexa­ne/methyl­ene chloride as its 0.44 methyl­ene chloride solvate (Fig. 2 ▸). **1d** crystallizes as a racemic mixture of two helimers in the monoclinic space group *C*2/*c*, and its structure is generally in line with that of the other three free base morpholino­chlorins that have been structurally described (Fig. 1 ▸): the *meso*-tolyl derivative **1b** with two eth­oxy substit­uents in the 2,3 positions of the morpholine (McCarthy *et al.*, 2003 ▸); the *meso*-phenyl derivative **1e** with a single meth­oxy substituent (Brückner *et al.*, 2011 ▸), and the *meso-*phenyl derivative **1f** lacking any morpholine substitution (Brückner *et al.*, 2011 ▸). The macrocycle in all morpholino­chlorins is non-planar. In the symmetrically substituted morpholino­chlorins, **1b**, **1d** and **1f**, individual mol­ecules are ruffled (Shelnutt *et al.*, 1998 ▸), feature a chiral axis and are helimeric. Derivative **1e** with only a single meth­oxy substit­uent on the morpholine (Brückner *et al.*, 2011 ▸) features a more saddled conformation of its macrocycle (Brückner *et al.*, 2011 ▸). The geometries of the morpholino rings also vary between the four structures. In the two 2,3-substituted derivatives, title compound **1d** and *meso*-tolyl derivative **1b**, the substituents are arranged *anti* to each other, and the morpholino rings adopt a conformation that is best described as half-twist. This stereoselective arrangement had been rationalized on steric and stereoelectronic grounds (Brückner *et al.*, 2011 ▸). The morpholine moiety in the mono-alk­oxy derivative **1e** adopts a half-boat conformation (Brückner *et al.*, 2011 ▸).

**Figure 3:**
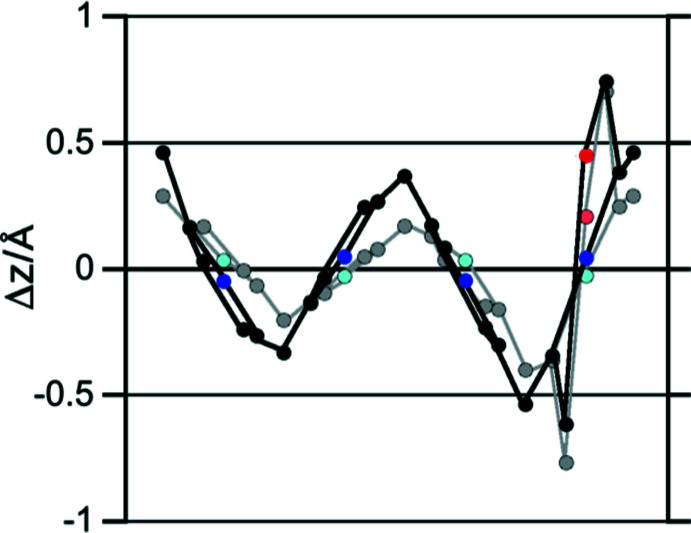
Out-of-plane displacement plots of macrocycles of the title compound **1d** (black trace) and morpholino­chlorin **1b** (gray trace).

Out-of-plane plots of the macrocycle conformations of **1b** and **1d** directly compare their ruffled conformation that allows the central nitro­gen atoms to remain idealized in the central plane (Fig. 3 ▸). The conformation of the C_20_N_4_O morpholino­chlorin macrocycle in **1d** is slightly more ruffled (r.m.s. = 0.323 Å) (Shelnutt *et al.*, 1998 ▸) than in **1b** (r.m.s. = 0.276 Å; Sharma *et al.*, 2017 ▸). While the tripyrrolic portion of **1d** is significantly more ruffled than the corresponding section of **1b**, the morpholine moieties are, except for the position of the ring oxygen, rather similar.

Similar to other *meso*-aryl porphyrinoids, the torsion angles in the morpholino­chlorins between the *meso*-aryl substituents and the mean plane of the macrocycle vary with the steric demand of the groups flanking the aryl substituents. The *meso*-penta­fluoro­phenyl groups neighboring the pyrrolic units (C_5_F_6_ rings of C27 and C33) face little steric constraints and adopt dihedral angles of 71.92 (2) and 74.95 (3)°, respectively. Those adjacent to the morpholine moiety (C_6_F_5_ rings of C21 and C39) are more sterically encumbered and are about 10° closer to perpendicular to the macrocycle plane, with values of 82.70 (3) and 81.44 (2)°. The corresponding values for compound **1b** are very similar, with values of 71.89 and 73.73°, and 89.55 and 86.32°, respectively.

**Figure 4:**
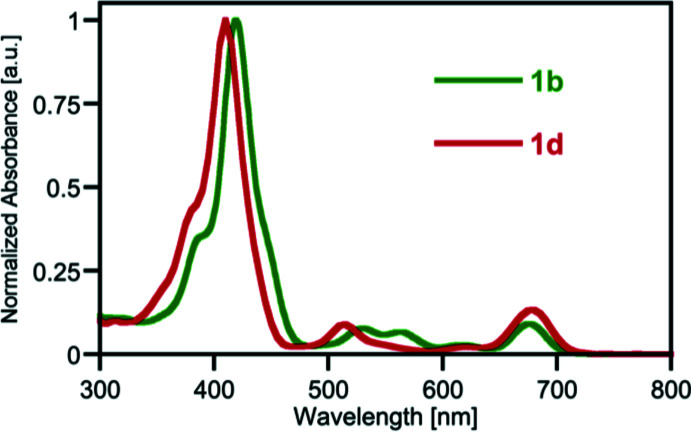
Normalized UV–vis spectra (CH_2_Cl_2_) of the compounds indicated.

The close structural relationship between the 2,3-disubstituted derivatives **1b** and **1d** allows us to investigate how minor conformational changes might affect the optical properties of the morpholino­chlorins. The torsion angles between the two C—C bonds in the morpholine units [C_a_—C_b_–(N)–C_b_—C_a_, C2—C1—(N1)—C4—C3 in **1d**] in the two morpholino­chlorins **1b** and **1d** vary slightly, with this angle being smaller in the title compound [35.2° in **1b** and 25.5 (4)° **1d**]. This angle is important as it strongly affects the λ_max_ of the morpholino­chlorins (Guberman-Pfeffer *et al.*, 2017 ▸), with a larger torsion angle being correlated to a longer λ_max_ in their UV–vis absorption spectra. However, while the UV–vis spectra of the two species show distinct differences, their λ_max_ values are essentially the same (680 nm in **1d** *vs* 678 nm in **1b**; Fig. 4 ▸), likely as the result of the combination of their differing conformation and electron-withdrawing natures of their *meso*-substituents (phenyl in **1b** and penta­fluoro­phenyl in **1d**).

## Supra­molecular features   

Inter­actions involving fluorine atoms play a dominant role in facilitating the arrangement of mol­ecules of **1d** in the crystal. Dominant are C—H⋯F hydrogen-bond-like inter­actions, involving both methyl as well as pyrrole moieties as the hydrogen-atom donor. Weak C—H⋯F inter­actions involving the solvent are also present. The most prominent of these inter­actions are given in Table 1 ▸ and are discussed below. Also present are a number of short fluorine⋯fluorine contacts, C—F⋯π inter­actions (towards the π system of a the macrocycle), and one severely slipped π-stacking inter­action between two penta­fluoro­phenyl rings.

**Figure 5:**
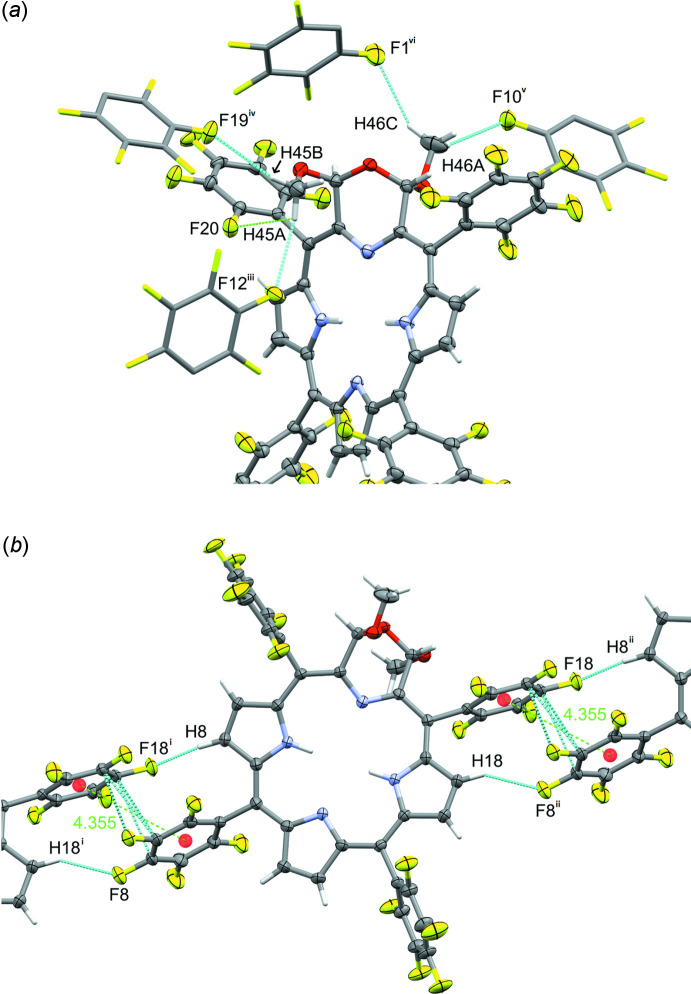
(*a*) C—H⋯F inter­actions involving the meth­oxy hydrogen atoms (turquoise dashed lines). Accepting moieties are truncated to their penta­fluoro­phenyl groups, and symmetry-related atoms not directly involved in an inter­action are shown in stick mode for clarity. (*b*) C—H⋯F and slipped π–π stacking inter­actions (turquoise dashed lines) connecting mol­ecules into infinite chains. Red spheres indicate the centroids of the respective aromatic rings, green dashed lines the distance between centroids (in Å). For symmetry codes, see Table 1 ▸.

The most prominent C—H⋯F inter­actions (Levina *et al.*, 2019 ▸) involve the two methyl groups of the 2,3-di­meth­oxy­morpholino unit (Fig. 5 ▸ *a*). Both meth­oxy substituents are engaged in several of these inter­actions: C45 exhibits inter­actions with fluorine atoms from three different penta­fluoro­phenyl groups: with meta fluorine atoms F12^iii^ and F19^iv^ [symmetry codes: (iii) −*x* + 1, *y*, −*z* + 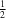; (iv) −*x* + 1, −*y* + 1, −*z* + 1], and one intra­molecular inter­action with F20, an *ortho*-fluorine atom. Angular and H⋯F distance values for this intra­molecular inter­action appear quite unfavorable: the C—H⋯F angle is only 103°, and the H⋯F distance is 2.82 Å. However, only a slight rotation of the methyl H atoms is required to create a much more favorable geometry, and the C⋯F distance between C45 and F20 is at 3.184 (3) quite short (the shortest of all C—H⋯F inter­actions observed in **1d**). Inter­actions involving the meth­oxy group of C46 involve F10^v^ and F1^vi^, two *ortho*-fluorine atoms [symmetry codes: (v) *x* − 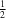, *y* − 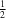, *z*; (vi) −*x* + 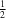, −*y* + 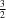, −*z* + 1]. Two C—H⋯F inter­actions originate from pyrrole moieties, involving H atoms at the pyrrole moieties flanking the morpholine unit: H8 towards F18^i^, and H18 towards F8^ii^, with both F8 and F18 being *para*-fluorine atoms [symmetry codes: (i) *x*, *y* + 1, *z*; (ii) *x*, *y* − 1, *z*]. These two inter­actions work in tandem with each other and with a severely slipped π–π stacking inter­action, between the rings of F6–F10 and F16^i^–F20^i^, connecting two opposite ends of the morpholino­chlorin mol­ecule with its neighbors to create infinite chains connected *via* C—H⋯F and slipped π–π stacking inter­actions (Fig. 5 ▸ *b*). The centroid-to-centroid distance of the π-stacking inter­action is 4.3551 (15) Å, with a ring slippage of 2.795 Å and a centroid-to-mean-plane distance of 3.1661 (12) Å. The last C—H⋯F inter­action involves the methyl­ene group of the minor moiety solvate methyl­ene chloride mol­ecule. Given the degree of disorder of the solvate mol­ecules (see *Refinement* section), this inter­action is probably vaguely defined at best and will not be discussed in detail.

**Figure 6:**
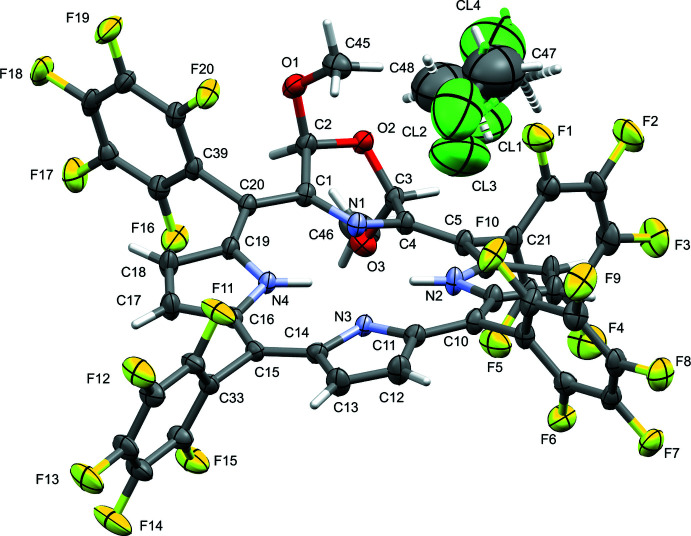
F⋯F inter­actions (turquoise dashed lines) creating a triangular motif. Symmetry-related moieties are truncated to their penta­fluoro­phenyl groups, and atoms not directly involved in an inter­action are shown in stick mode for clarity. Symmetry codes: (vii) −*x* + 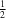, *y* − 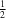, −*z* + 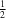; (viii) −*x* + 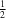, −*y* + 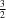, −*z* + 1.

**Figure 7:**
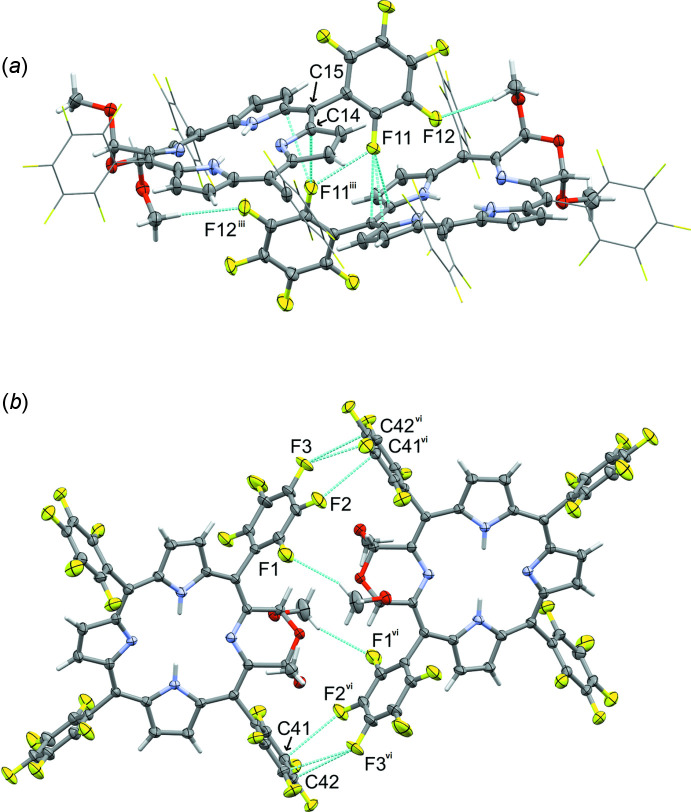
F⋯F, C—F⋯π and C—H⋯F inter­actions (turquoise dashed lines) connecting mol­ecules into dimers. Penta­fluoro­phenyl groups not involved in the shown inter­actions are shown in wireframe mode for clarity. For symmetry codes, see Table 1 ▸.

Besides C—H⋯F inter­actions, which are generally considered as directional inter­actions similar in strength to the better investigated C—H⋯O inter­actions, **1d** also features a number of short F⋯F contacts. In contrast to halogen⋯halogen bonds involving chlorine, and especially bromine and iodine (the classical halogen bonds), inter­actions between two fluorine atoms are different and much weaker in nature (Cavallo *et al.*, 2016 ▸). C—F⋯F—C inter­actions are generally not directional and do usually not play any structure-directing role. The energy of inter­molecular C—F⋯F—C inter­actions in mol­ecular compounds is estimated at <4 kJ mol^−1^, substanti­ally lower that of C—H⋯F inter­actions, which tend to range from 5 to 7 kJ mol^−1^. They are, however, still regarded as weakly attractive and contributing to the overall stability of the packing arrangement (Levina *et al.*, 2019 ▸). Three distinct inter­actions of this kind with F⋯F distances under 3.0 Å are observed in **1d**. Fluorine atom F5 forms close contacts with F7 and F8 located at the C_6_F_5_ ring of a neighboring mol­ecule. The F⋯F distances are 2.797 (2) Å (F5⋯F7^vii^) and 2.828 (3) Å (F5⋯F8^vii^) [symmetry code: (vii) 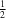 − *x*, −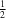 + *y*, 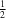 − *z*]. With the intra­molecular distance between F7 and F8 being 2.726 (2) Å, this leads to the formation of a nearly equilateral triangle of F atoms (Fig. 6 ▸ *a*). It should be noted that atom F8 of this F_3_-triangle also acts as the acceptor of the C18—H18⋯F8^ii^ contact and the backside of the aromatic ring of F8 is involved in the slipped π–π stacking inter­action (see discussion above). Fluorine atom F11 features a close contact with a symmetry-created copy of itself, created by a twofold axis. The F⋯F^iii^ distance here is 2.783 (3) Å, and the C—F⋯F^iii^ angle is 125.5 (2)° [symmetry code: (iii) −*x* + 1, *y*, −*z* + 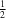]. F11 also inter­acts with the π system of the macrocycle created by the same twofold axis, with F⋯C distances towards C14^iii^ and C15^iii^ of 3.046 (3) and 3.035 (3) Å, and F12 acts as the acceptor of the C45—H45*C*⋯F12^iii^ inter­action, thus creating a larger multi-inter­action contact between the two neighboring mol­ecules with mutually stabilizing inter­actions (Fig. 7 ▸ *a*). The last clearly recognizable inter­action between fluorine atoms is an inversion-symmetric pair of two F⋯F contacts, involving F14 and F15 of one C_6_F_5_ ring and their symmetry-related counterparts across a crystallographic inversion center (Fig. 6 ▸ *b*). The F14⋯F15^viii^ distance is 7.248 (2) Å. The C37—F14⋯F15^viii^ angle here is 168.6 (2)° [symmetry code: (viii) 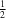 − *x*, 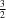 − *y*, −*z*].

Besides F11, F2 and F3 are also involved in inter­molecular C—F⋯π inter­actions, pointing nearly perpendicularly towards C atoms (C41^vi^ and C42^vi^) of another penta­fluoro­phenyl ring [symmetry code: (vi) −*x* + 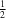, −*y* + 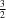, −*z* + 1]. The F⋯C distances are 3.034 (3) and 2.978 (3) Å for F2 and F3, respectively. There are two inter­actions of this kind per mol­ecule, one as the C—F donor and one as the π-density moiety accepting the C—F⋯π bond, connecting mol­ecules into centrosymmetric dimers. One of the methyl C—H⋯F contacts (towards F1) is also involved in the formation of these dimers (Fig. 7 ▸ *b*).

## Database survey   

A CSD search (Version 5.41 with updates up to May 2020; Groom *et al.*, 2016 ▸) for porphyrinic macrocyles of three pyrroles and a single six-membered ring while retaining the porphyrin-like architecture of four central nitro­gen atoms reveals 24 structures: six pyriporphyrins (*i.e*., porphyrinoids containing a pyridine building block), fifteen morpholino­chlorins, two thio­morpholines [UCIKOJ and UCILIE (Sharma *et al.*, 2016 ▸)], and a single 1,3-oxazinochlorin (WUDMIT; Meehan *et al.*, 2015 ▸). Among the 1,4-morpholino­chlorins, six are free base structures, the remainder are metal complexes [of Cu^II^, Ni^II^ – most frequently, Zn^II^, Ag^II^ and Pd^II^, see Fig. 1 ▸ for CSD codes]. Only a single structure, (**1b**, RUXJUP; McCarthy *et al.*, 2003 ▸) is directly comparable to **1d**; all the others contain either covalent morpholine-to-*meso*-aryl linkages (AVICAK; Daniell & Brückner, 2004 ▸; Brückner *et al.*, 2011 ▸), a reduced pyrrole moiety (KECFEF; Samankumara *et al.*, 2012 ▸; Guberman-Pfeffer *et al.*, 2017 ▸), or both (KECDUT; Samankumara *et al.*, 2012 ▸; Guberman-Pfeffer *et al.*, 2017 ▸), or only one (**1e**, AVICEO; Brückner *et al.*, 2011 ▸) or no alk­oxy substituents (**1f**, AVICOY; Brückner *et al.*, 2011 ▸). These structural factors affect the conformation of the chromophore of evidently large plasticity (Brückner *et al.*, 2011 ▸; Guberman-Pfeffer *et al.*, 2017 ▸; Sharma *et al.*, 2017 ▸).

## Synthesis and crystallization   

We prepared the title compound **1d** according to an established strategy from the corresponding 2,3-di­hydroxy­chlorin **2** (Fig. 1 ▸) (Brückner *et al.*, 2011 ▸): Oxidative diol cleavage is followed, in a one-pot approach, by a nucleophile-induced (methanol), acid-catalyzed intra­molecular ring closure and subsequent double-acetalization. Specifically, *meso*-tetra­kis­(penta­fluoro­phen­yl)-2,3-di­hydroxy­chlorin **2** (Hyland *et al.*, 2012 ▸) (30 mg, 2.97 × 10 ^−5^ mol) was dissolved in a 50 mL two-necked round-bottom flask equipped with a stir bar and gas in/outlets in CHCl_3_ (7 mL, amylene stabilized). The vessel was put under a protective atmosphere of N_2_. Freshly prepared NaIO_4_ heterogenized on silica (Zhong & Shing, 1997 ▸) (0.30 g) and dry MeOH (∼0.3 mL) were added and the reaction was acidified with the vapors from a conc. aqueous HCl bottle (36%), delivered to the surface of the solution as puffs (3 × ∼1 mL) from a Pasteur pipette topped by a small latex bulb. The reaction was shielded from light by aluminum foil, stirred at ambient temperature and monitored by TLC (silica gel/CH_2_Cl_2_). After 24 h reaction time, no further reaction was observed; the solution was filtered (glass frit M) and the filtrate reduced to dryness by rotary evaporation. The crude product was dissolved in CH_2_Cl_2_ (∼1 mL), loaded onto a preparative TLC plate (500 µm silica gel, 10 × 20 cm) that was developed with a 1:1 CH_2_Cl_2_:hexane mixture as eluent. The main brown band was retrieved, ground into a fine powder, and extracted in a cotton-plugged small column with CH_2_Cl_2_. The addition of ∼20 vol% MeOH to the filtrate and slow removal of the CH_2_Cl_2_ by rotary evaporation precipitated the product, which could be isolated by filtration (Kontes microfiltration setup). After vacuum-drying at ambient temperature, **1d** was retrieved as a dark-purple powder in 66% yield (21 mg). MW = 1052.63 g mol^−1^; ^1^H NMR (300 MHz, CDCl_3_, 300 K): δ 8.62 (*d*, *J* = 4.9 Hz, 2H), 8.42 (*s*, 2H), 8.28 (*d*, *J* = 5.4 Hz, 2H), 6.56 (*s*, 2H), 3.08 (*s*, 6H), −1.09 (*s*, 2H). UV–vis (CH_2_Cl_2_) λ_max_ nm (log e): 410 (5.20), 515 (4.16), 620 (3.50), 680 (4.33). HR–MS (ESI+, 100% CH_3_CN, TOF): *m/z* calculated for C_46_H_16_F_20_N_4_O_3_ (*M*H^+^) 1053.0976; found 1053.0904 (error: 7 ppm). Single crystals suitable for X-ray diffraction were grown in the dark by slow vapor diffusion of hexane into a solution of **1d** in CH_2_Cl_2_.

## Refinement   

Crystal data, data collection and structure refinement details are summarized in Table 2 ▸.

Crystals for diffraction analysis were taken directly out of the mother liquor (methyl­ene chloride/hexa­ne), mounted immediately on a MiTeGen micromesh mount with the help of a trace of Fomblin oil (a perfluorinated ether), and flash cooled in the cold stream of the diffractometer. Over several hours, no desolvation was observed for crystals remaining immersed in Fomblin oil on the crystal mounting microscope slide.

The solvate methyl­ene chloride mol­ecule is disordered over four positions around an inversion center (each two being symmetry equivalent). The C—Cl and Cl⋯Cl distances were restrained to target values and *U* ^ij^ components of ADPs for disordered atoms closer to each other than 2.0 Å were restrained to be similar. Occupancies of each of the two symmetry-equivalent sites were freely refined, resulting in a total occupancy slightly below unity [two × 0.241 (5) and two × 0.199 (4), for a total site occupancy of 88%]. Disorder with hexane, the other type of solvent used during crystallization, was excluded as a possibility due to the limited size of the solvate pocket, and it is thus assumed that 12% of void spaces in the crystal structure remained unoccupied during the crystallization process.

N-bound H atoms were located in a difference electron-density map and were freely refined. H atoms attached to carbon atoms were positioned geometrically and constrained to ride on their parent atoms. C—H bond distances were constrained to 0.95 Å for pyrrole CH moieties, and to 1.00, 0.99 and 0.98 Å for aliphatic CH, CH_2_ and CH_3_ moieties, respectively. Methyl CH_3_ groups were allowed to rotate but not to tip to best fit the experimental electron density. *U*_iso_(H) values were set to a multiple of *U*_eq_(C) with 1.5 for CH_3_, and 1.2 for CH and CH_2_ units, respectively.

## Supplementary Material

Crystal structure: contains datablock(s) I, global. DOI: 10.1107/S2056989020009093/is5543sup1.cif

Structure factors: contains datablock(s) I. DOI: 10.1107/S2056989020009093/is5543Isup2.hkl

CCDC reference: 2013871

Additional supporting information:  crystallographic information; 3D view; checkCIF report

## Figures and Tables

**Table 1:** Hydrogen-bond geometry (Å, °)

*D*—H⋯*A*	*D*—H	H⋯*A*	*D*⋯*A*	*D*—H⋯*A*
C8—H8⋯F18^i^	0.95	2.59	3.424 (3)	147
C18—H18⋯F8^ii^	0.95	2.51	3.319 (3)	143
C45—H45*A*⋯F12^iii^	0.98	2.55	3.519 (3)	169
C45—H45*B*⋯F19^iv^	0.98	2.66	3.389 (3)	132
C45—H45*A*⋯F20	0.98	2.82	3.184 (3)	103
C46—H46*A*⋯F10^v^	0.98	2.63	3.496 (4)	148
C46—H46*C*⋯F1^vi^	0.98	2.60	3.505 (4)	153
C48—H48*B*⋯F12^iii^	0.99	2.42	3.25 (5)	141
N2—H2*A*⋯N1	0.85 (3)	2.56 (3)	3.040 (3)	117 (3)
N2—H2*A*⋯N3	0.85 (3)	2.33 (3)	2.858 (3)	121 (3)
N4—H4⋯N1	0.79 (3)	2.61 (3)	3.040 (3)	116 (2)
N4—H4⋯N3	0.79 (3)	2.33 (3)	2.852 (3)	125 (3)

Symmetry codes: (i) 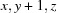; (ii) 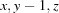; (iii) 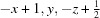; (iv) 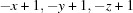; (v) 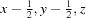; (vi) 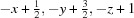.

**Table 2:** Experimental details

Crystal data	
Chemical formula	C_46_H_16_F_20_N_4_O_3_·0.44CH_2_Cl_2_
*M*_r_	1090.00
Crystal system, space group	Monoclinic, *C*2/*c*
Temperature (K)	150
*a*, *b*, *c* (Å)	23.1774 (10), 15.4767 (7), 26.0442 (13)
β (°)	113.1683 (18)
*V* (Å^3^)	8588.9 (7)
*Z*	8
Radiation type	Mo *K*α
μ (mm^−1^)	0.22
Crystal size (mm)	0.11 × 0.08 × 0.07

Data collection	
Diffractometer	Bruker D8 Quest CMOS
Absorption correction	Multi-scan (*SADABS*; Krause *et al.*, 2015 ▸)
*T*_min_, *T*_max_	0.671, 0.746
No. of measured, independent and observed [*I* > 2σ(*I*)] reflections	52998, 10495, 6698
*R*_int_	0.082
(sin θ/λ)_max_ (Å^−1^)	0.667

Refinement	
*R*[*F* ^2^ > 2σ(*F* ^2^)], *wR*(*F* ^2^), *S*	0.058, 0.164, 1.03
No. of reflections	10495
No. of parameters	724
No. of restraints	66
H-atom treatment	H atoms treated by a mixture of independent and constrained refinement
Δρ_max_, Δρ_min_ (e Å^−3^)	0.84, −0.46

Computer programs: *APEX3* and *SAINT* (Bruker, 2016 ▸), *SHELXS97* (Sheldrick, 2008 ▸), *SHELXL2018/3* (Sheldrick, 2015 ▸), *shelXle* (Hübschle *et al.*, 2011 ▸), *Mercury* (Macrae *et al.*, 2020 ▸), *publCIF* (Westrip, 2010 ▸) and *PLATON* (Spek, 2020 ▸).

## supplementary crystallographic information

### Crystal data

**Table:**

C_46_H_16_F_20_N_4_O_3_·0.44CH_2_Cl_2_	*F*(000) = 4340
*M**_r_* = 1090.00	*D*_x_ = 1.686 Mg m^−^^3^
Monoclinic, *C*2/*c*	Mo *K*α radiation, λ = 0.71073 Å
*a* = 23.1774 (10) Å	Cell parameters from 9923 reflections
*b* = 15.4767 (7) Å	θ = 3.0–28.3°
*c* = 26.0442 (13) Å	µ = 0.22 mm^−^^1^
β = 113.1683 (18)°	*T* = 150 K
*V* = 8588.9 (7) Å^3^	Block, brown
*Z* = 8	0.11 × 0.08 × 0.07 mm

### Data collection

**Table:**

Bruker D8 Quest CMOS diffractometer	10495 independent reflections
Radiation source: sealed tube X-ray source	6698 reflections with *I* > 2σ(*I*)
Triumph curved graphite crystal monochromator	*R*_int_ = 0.082
ω and phi scans	θ_max_ = 28.3°, θ_min_ = 3.0°
Absorption correction: multi-scan (*SADABS*; Krause *et al*., 2015)	*h* = −30→28
*T*_min_ = 0.671, *T*_max_ = 0.746	*k* = −19→20
52998 measured reflections	*l* = −32→34

### Refinement

**Table:**

Refinement on *F*^2^	Primary atom site location: structure-invariant direct methods
Least-squares matrix: full	Secondary atom site location: difference Fourier map
*R*[*F*^2^ > 2σ(*F*^2^)] = 0.058	Hydrogen site location: mixed
*wR*(*F*^2^) = 0.164	H atoms treated by a mixture of independent and constrained refinement
*S* = 1.03	*w* = 1/[σ^2^(*F*_o_^2^) + (0.0789*P*)^2^ + 8.847*P*] where *P* = (*F*_o_^2^ + 2*F*_c_^2^)/3
10495 reflections	(Δ/σ)_max_ < 0.001
724 parameters	Δρ_max_ = 0.84 e Å^−^^3^
66 restraints	Δρ_min_ = −0.46 e Å^−^^3^

### Special details

**Table:**

Geometry. All esds (except the esd in the dihedral angle between two l.s. planes) are estimated using the full covariance matrix. The cell esds are taken into account individually in the estimation of esds in distances, angles and torsion angles; correlations between esds in cell parameters are only used when they are defined by crystal symmetry. An approximate (isotropic) treatment of cell esds is used for estimating esds involving l.s. planes.

### Fractional atomic coordinates and isotropic or equivalent isotropic displacement parameters (Å^2^)

**Table:**

	*x*	*y*	*z*	*U*_iso_*/*U*_eq_	Occ. (<1)
C1	0.32635 (11)	0.72878 (15)	0.38040 (10)	0.0219 (5)	
C2	0.32104 (12)	0.68340 (16)	0.43114 (10)	0.0255 (5)	
H2	0.296174	0.629244	0.417471	0.031*	
C3	0.24577 (11)	0.79201 (15)	0.42231 (10)	0.0233 (5)	
H3	0.229375	0.828639	0.445218	0.028*	
C4	0.27686 (11)	0.85048 (15)	0.39362 (10)	0.0227 (5)	
C5	0.26559 (12)	0.93899 (15)	0.39395 (11)	0.0247 (5)	
C6	0.29046 (12)	1.00692 (16)	0.37303 (11)	0.0272 (5)	
C7	0.28844 (13)	1.09651 (16)	0.38542 (12)	0.0321 (6)	
H7	0.268250	1.120434	0.407602	0.039*	
C8	0.32024 (13)	1.14180 (16)	0.36019 (12)	0.0313 (6)	
H8	0.327240	1.202425	0.362572	0.038*	
C9	0.34121 (12)	1.08218 (15)	0.32952 (11)	0.0268 (5)	
C10	0.37142 (11)	1.10000 (15)	0.29387 (11)	0.0256 (5)	
C11	0.38331 (11)	1.03995 (15)	0.25895 (10)	0.0236 (5)	
C12	0.40467 (13)	1.06396 (17)	0.21565 (12)	0.0324 (6)	
H12	0.417330	1.119836	0.209104	0.039*	
C13	0.40309 (13)	0.99144 (17)	0.18644 (11)	0.0321 (6)	
H13	0.413110	0.986312	0.154535	0.039*	
C14	0.38299 (11)	0.92292 (15)	0.21356 (10)	0.0231 (5)	
C15	0.37907 (11)	0.83510 (15)	0.19838 (10)	0.0215 (5)	
C16	0.36755 (11)	0.76756 (15)	0.22787 (10)	0.0226 (5)	
C17	0.37311 (13)	0.67770 (16)	0.21961 (11)	0.0318 (6)	
H17	0.380112	0.652305	0.189355	0.038*	
C18	0.36671 (13)	0.63440 (16)	0.26236 (12)	0.0323 (6)	
H18	0.369061	0.573502	0.267333	0.039*	
C19	0.35586 (11)	0.69561 (15)	0.29864 (10)	0.0231 (5)	
C20	0.34768 (11)	0.67585 (14)	0.34804 (10)	0.0214 (5)	
C21	0.22315 (12)	0.97069 (15)	0.42126 (11)	0.0271 (5)	
C22	0.24512 (13)	0.99038 (17)	0.47767 (12)	0.0320 (6)	
C23	0.20658 (14)	1.02359 (18)	0.50208 (12)	0.0360 (6)	
C24	0.14432 (15)	1.0389 (2)	0.46966 (13)	0.0405 (7)	
C25	0.12069 (14)	1.0214 (2)	0.41347 (13)	0.0417 (7)	
C26	0.16036 (13)	0.98641 (18)	0.39028 (12)	0.0333 (6)	
C27	0.38931 (12)	1.19202 (15)	0.29033 (11)	0.0256 (5)	
C28	0.34468 (12)	1.25553 (16)	0.26532 (10)	0.0263 (5)	
C29	0.36086 (13)	1.34043 (16)	0.26142 (11)	0.0287 (5)	
C30	0.42335 (13)	1.36344 (15)	0.28171 (11)	0.0300 (6)	
C31	0.46876 (13)	1.30227 (18)	0.30576 (13)	0.0338 (6)	
C32	0.45121 (12)	1.21796 (16)	0.30977 (12)	0.0299 (6)	
C33	0.39339 (11)	0.81235 (15)	0.14862 (10)	0.0229 (5)	
C34	0.45380 (11)	0.81801 (17)	0.15011 (10)	0.0282 (5)	
C35	0.46780 (12)	0.79981 (18)	0.10433 (11)	0.0313 (6)	
C36	0.42068 (13)	0.77496 (19)	0.05528 (11)	0.0337 (6)	
C37	0.36041 (13)	0.7670 (2)	0.05249 (11)	0.0365 (6)	
C38	0.34730 (12)	0.78505 (17)	0.09886 (11)	0.0301 (6)	
C39	0.36233 (11)	0.58285 (15)	0.36504 (10)	0.0219 (5)	
C40	0.31708 (12)	0.51876 (16)	0.34665 (11)	0.0270 (5)	
C41	0.33125 (13)	0.43302 (16)	0.36056 (11)	0.0291 (6)	
C42	0.39189 (14)	0.41008 (15)	0.39319 (11)	0.0311 (6)	
C43	0.43823 (13)	0.47182 (17)	0.41231 (11)	0.0302 (6)	
C44	0.42281 (11)	0.55716 (15)	0.39797 (11)	0.0254 (5)	
C45	0.42137 (13)	0.7312 (2)	0.49423 (12)	0.0377 (6)	
H45A	0.431108	0.757883	0.464446	0.057*	
H45B	0.460179	0.710236	0.523699	0.057*	
H45C	0.401672	0.774104	0.509843	0.057*	
C46	0.15720 (16)	0.7051 (3)	0.40359 (14)	0.0538 (9)	
H46A	0.119227	0.684073	0.373152	0.081*	
H46B	0.145443	0.743803	0.427672	0.081*	
H46C	0.180923	0.656005	0.425570	0.081*	
N1	0.31243 (9)	0.81349 (13)	0.36912 (9)	0.0229 (4)	
N2	0.32301 (10)	1.00102 (14)	0.33927 (9)	0.0263 (5)	
H2A	0.3274 (14)	0.954 (2)	0.3242 (13)	0.039 (8)*	
N3	0.37108 (9)	0.95377 (12)	0.25756 (8)	0.0210 (4)	
N4	0.35551 (9)	0.77493 (13)	0.27530 (9)	0.0211 (4)	
H4	0.3538 (13)	0.8197 (19)	0.2894 (12)	0.028 (8)*	
O1	0.37925 (9)	0.66032 (12)	0.47187 (8)	0.0325 (4)	
O2	0.29008 (8)	0.73396 (11)	0.45788 (7)	0.0258 (4)	
O3	0.19528 (8)	0.75125 (12)	0.38073 (8)	0.0309 (4)	
F1	0.30561 (9)	0.97758 (13)	0.50994 (7)	0.0508 (5)	
F2	0.23007 (9)	1.04108 (13)	0.55689 (7)	0.0536 (5)	
F3	0.10730 (9)	1.07298 (14)	0.49265 (8)	0.0587 (5)	
F4	0.06062 (9)	1.03781 (17)	0.38111 (9)	0.0701 (7)	
F5	0.13564 (8)	0.97084 (13)	0.33525 (7)	0.0483 (5)	
F6	0.28370 (7)	1.23508 (10)	0.24473 (7)	0.0360 (4)	
F7	0.31630 (7)	1.40025 (10)	0.23865 (7)	0.0377 (4)	
F8	0.43987 (8)	1.44566 (10)	0.27921 (8)	0.0423 (4)	
F9	0.52959 (8)	1.32485 (11)	0.32567 (9)	0.0519 (5)	
F10	0.49729 (7)	1.15998 (10)	0.33406 (8)	0.0446 (4)	
F11	0.50098 (7)	0.84265 (13)	0.19692 (6)	0.0448 (4)	
F12	0.52687 (7)	0.80701 (13)	0.10779 (7)	0.0463 (4)	
F13	0.43327 (8)	0.75890 (13)	0.01025 (7)	0.0493 (5)	
F14	0.31452 (8)	0.74177 (15)	0.00464 (7)	0.0586 (6)	
F15	0.28789 (7)	0.77668 (12)	0.09439 (7)	0.0429 (4)	
F16	0.25774 (7)	0.54003 (10)	0.31493 (7)	0.0408 (4)	
F17	0.28647 (8)	0.37195 (10)	0.34199 (8)	0.0424 (4)	
F18	0.40620 (9)	0.32672 (9)	0.40659 (7)	0.0430 (4)	
F19	0.49691 (8)	0.44860 (11)	0.44487 (8)	0.0477 (5)	
F20	0.46884 (7)	0.61620 (9)	0.41722 (7)	0.0355 (4)	
C47	0.4785 (12)	0.980 (4)	0.4881 (10)	0.154 (6)	0.241 (5)
H47A	0.476482	0.922171	0.470843	0.184*	0.241 (5)
H47B	0.454112	1.021606	0.458893	0.184*	0.241 (5)
Cl1	0.5576 (3)	1.0144 (6)	0.5239 (4)	0.160 (4)	0.241 (5)
Cl2	0.4511 (5)	0.9757 (6)	0.5439 (5)	0.144 (3)	0.241 (5)
C48	0.508 (3)	0.962 (3)	0.4834 (13)	0.142 (7)	0.199 (4)
H48A	0.553867	0.953404	0.498144	0.171*	0.199 (4)
H48B	0.489108	0.905124	0.468872	0.171*	0.199 (4)
Cl3	0.4912 (6)	1.0246 (8)	0.4255 (4)	0.127 (4)	0.199 (4)
Cl4	0.4918 (11)	0.9784 (13)	0.5413 (7)	0.204 (6)	0.199 (4)

### Atomic displacement parameters (Å^2^)

**Table:**

	*U*^11^	*U*^22^	*U*^33^	*U*^12^	*U*^13^	*U*^23^
C1	0.0229 (11)	0.0192 (11)	0.0273 (12)	−0.0013 (9)	0.0139 (10)	0.0007 (9)
C2	0.0324 (13)	0.0225 (12)	0.0275 (13)	0.0032 (10)	0.0180 (11)	0.0033 (10)
C3	0.0278 (12)	0.0205 (11)	0.0256 (12)	0.0020 (9)	0.0150 (10)	−0.0001 (9)
C4	0.0286 (12)	0.0208 (11)	0.0240 (12)	0.0006 (9)	0.0162 (10)	0.0005 (9)
C5	0.0317 (12)	0.0213 (12)	0.0291 (13)	0.0020 (10)	0.0206 (11)	0.0003 (10)
C6	0.0349 (13)	0.0215 (12)	0.0341 (14)	0.0035 (10)	0.0232 (11)	0.0009 (10)
C7	0.0477 (16)	0.0201 (12)	0.0413 (16)	0.0035 (11)	0.0312 (13)	−0.0032 (11)
C8	0.0456 (15)	0.0173 (12)	0.0409 (15)	0.0003 (11)	0.0276 (13)	−0.0024 (11)
C9	0.0332 (13)	0.0195 (12)	0.0341 (14)	−0.0005 (10)	0.0202 (11)	−0.0006 (10)
C10	0.0304 (13)	0.0189 (11)	0.0321 (14)	−0.0009 (10)	0.0174 (11)	0.0009 (10)
C11	0.0264 (12)	0.0220 (12)	0.0266 (13)	0.0007 (10)	0.0149 (10)	0.0025 (10)
C12	0.0484 (16)	0.0232 (13)	0.0366 (15)	−0.0022 (11)	0.0286 (13)	0.0029 (11)
C13	0.0486 (16)	0.0278 (13)	0.0309 (14)	−0.0008 (12)	0.0273 (13)	0.0019 (11)
C14	0.0268 (12)	0.0232 (12)	0.0216 (12)	0.0021 (10)	0.0121 (10)	0.0029 (9)
C15	0.0233 (11)	0.0243 (12)	0.0182 (11)	0.0027 (9)	0.0097 (9)	−0.0012 (9)
C16	0.0258 (11)	0.0221 (11)	0.0217 (12)	0.0008 (9)	0.0112 (10)	−0.0023 (9)
C17	0.0474 (16)	0.0232 (13)	0.0322 (14)	0.0019 (11)	0.0238 (13)	−0.0030 (11)
C18	0.0511 (16)	0.0186 (12)	0.0359 (15)	0.0031 (11)	0.0263 (13)	−0.0012 (11)
C19	0.0278 (12)	0.0170 (11)	0.0264 (13)	0.0023 (9)	0.0128 (10)	0.0005 (9)
C20	0.0240 (11)	0.0171 (11)	0.0242 (12)	0.0004 (9)	0.0108 (10)	0.0004 (9)
C21	0.0405 (14)	0.0181 (11)	0.0333 (14)	0.0028 (10)	0.0260 (12)	0.0015 (10)
C22	0.0390 (15)	0.0302 (13)	0.0330 (14)	0.0031 (11)	0.0209 (12)	0.0024 (11)
C23	0.0560 (18)	0.0325 (14)	0.0324 (15)	−0.0008 (13)	0.0314 (14)	−0.0034 (12)
C24	0.0498 (17)	0.0415 (16)	0.0476 (18)	0.0070 (14)	0.0377 (15)	−0.0023 (14)
C25	0.0423 (16)	0.0501 (18)	0.0452 (18)	0.0093 (14)	0.0306 (14)	−0.0005 (14)
C26	0.0436 (16)	0.0339 (14)	0.0322 (15)	0.0060 (12)	0.0254 (13)	0.0010 (12)
C27	0.0355 (13)	0.0199 (12)	0.0301 (13)	−0.0014 (10)	0.0224 (11)	−0.0004 (10)
C28	0.0307 (12)	0.0275 (13)	0.0247 (13)	−0.0023 (10)	0.0152 (10)	0.0001 (10)
C29	0.0434 (15)	0.0195 (12)	0.0288 (13)	0.0045 (11)	0.0203 (12)	0.0036 (10)
C30	0.0466 (15)	0.0171 (11)	0.0339 (14)	−0.0043 (11)	0.0241 (12)	0.0004 (10)
C31	0.0326 (14)	0.0279 (14)	0.0460 (17)	−0.0065 (11)	0.0209 (12)	−0.0019 (12)
C32	0.0314 (13)	0.0223 (12)	0.0417 (16)	0.0025 (10)	0.0206 (12)	0.0016 (11)
C33	0.0297 (12)	0.0215 (11)	0.0196 (11)	0.0028 (10)	0.0119 (10)	0.0010 (9)
C34	0.0268 (12)	0.0361 (14)	0.0201 (12)	0.0006 (11)	0.0074 (10)	−0.0036 (10)
C35	0.0271 (12)	0.0414 (15)	0.0294 (14)	0.0032 (11)	0.0156 (11)	−0.0019 (12)
C36	0.0384 (14)	0.0458 (16)	0.0228 (13)	0.0008 (12)	0.0183 (11)	−0.0065 (12)
C37	0.0350 (14)	0.0501 (17)	0.0222 (13)	−0.0022 (13)	0.0088 (11)	−0.0097 (12)
C38	0.0268 (12)	0.0374 (15)	0.0266 (13)	−0.0001 (11)	0.0110 (11)	−0.0016 (11)
C39	0.0296 (12)	0.0176 (11)	0.0241 (12)	0.0027 (9)	0.0165 (10)	0.0005 (9)
C40	0.0301 (13)	0.0240 (12)	0.0301 (13)	0.0002 (10)	0.0153 (11)	0.0000 (10)
C41	0.0421 (15)	0.0210 (12)	0.0318 (14)	−0.0065 (11)	0.0227 (12)	−0.0039 (10)
C42	0.0544 (17)	0.0161 (12)	0.0323 (14)	0.0055 (11)	0.0272 (13)	0.0055 (10)
C43	0.0356 (14)	0.0252 (13)	0.0303 (14)	0.0103 (11)	0.0135 (11)	0.0049 (11)
C44	0.0307 (13)	0.0192 (12)	0.0314 (14)	0.0004 (10)	0.0178 (11)	−0.0003 (10)
C45	0.0331 (14)	0.0481 (17)	0.0335 (15)	−0.0003 (13)	0.0148 (12)	0.0001 (13)
C46	0.0450 (17)	0.076 (2)	0.0431 (19)	−0.0305 (17)	0.0199 (15)	−0.0035 (17)
N1	0.0264 (10)	0.0187 (9)	0.0280 (11)	0.0008 (8)	0.0152 (9)	−0.0009 (8)
N2	0.0383 (12)	0.0163 (10)	0.0344 (12)	−0.0005 (9)	0.0251 (10)	−0.0020 (9)
N3	0.0259 (10)	0.0159 (9)	0.0242 (10)	0.0022 (8)	0.0129 (8)	0.0002 (8)
N4	0.0273 (10)	0.0157 (10)	0.0237 (11)	0.0018 (8)	0.0138 (8)	−0.0008 (8)
O1	0.0378 (10)	0.0317 (10)	0.0316 (10)	0.0101 (8)	0.0175 (8)	0.0083 (8)
O2	0.0320 (9)	0.0261 (9)	0.0256 (9)	0.0062 (7)	0.0180 (7)	0.0042 (7)
O3	0.0312 (9)	0.0353 (10)	0.0300 (10)	−0.0064 (8)	0.0162 (8)	−0.0016 (8)
F1	0.0498 (10)	0.0691 (13)	0.0357 (10)	0.0102 (9)	0.0193 (8)	−0.0037 (9)
F2	0.0713 (13)	0.0666 (13)	0.0349 (10)	0.0013 (10)	0.0337 (9)	−0.0103 (9)
F3	0.0654 (12)	0.0743 (14)	0.0595 (12)	0.0142 (10)	0.0494 (11)	−0.0088 (10)
F4	0.0427 (10)	0.1148 (19)	0.0596 (13)	0.0267 (12)	0.0274 (10)	−0.0053 (12)
F5	0.0441 (10)	0.0728 (13)	0.0338 (9)	0.0099 (9)	0.0215 (8)	−0.0069 (9)
F6	0.0310 (8)	0.0342 (8)	0.0413 (9)	−0.0022 (7)	0.0125 (7)	0.0042 (7)
F7	0.0466 (9)	0.0273 (8)	0.0419 (9)	0.0083 (7)	0.0205 (8)	0.0107 (7)
F8	0.0551 (10)	0.0225 (8)	0.0540 (11)	−0.0077 (7)	0.0265 (9)	0.0035 (7)
F9	0.0364 (9)	0.0377 (10)	0.0837 (15)	−0.0099 (8)	0.0257 (9)	0.0041 (9)
F10	0.0337 (9)	0.0302 (8)	0.0734 (13)	0.0042 (7)	0.0248 (8)	0.0083 (8)
F11	0.0279 (8)	0.0814 (13)	0.0246 (8)	−0.0061 (8)	0.0096 (6)	−0.0144 (8)
F12	0.0317 (8)	0.0777 (13)	0.0365 (9)	−0.0014 (8)	0.0209 (7)	−0.0114 (9)
F13	0.0494 (10)	0.0786 (13)	0.0279 (9)	−0.0028 (9)	0.0238 (8)	−0.0149 (9)
F14	0.0414 (10)	0.1027 (17)	0.0276 (9)	−0.0118 (10)	0.0092 (8)	−0.0243 (10)
F15	0.0287 (8)	0.0689 (12)	0.0319 (9)	−0.0071 (8)	0.0127 (7)	−0.0102 (8)
F16	0.0297 (8)	0.0322 (8)	0.0525 (11)	−0.0023 (7)	0.0076 (7)	−0.0015 (7)
F17	0.0542 (10)	0.0242 (8)	0.0542 (11)	−0.0136 (7)	0.0271 (9)	−0.0056 (7)
F18	0.0712 (12)	0.0176 (7)	0.0433 (10)	0.0076 (7)	0.0259 (9)	0.0083 (7)
F19	0.0432 (10)	0.0335 (9)	0.0568 (12)	0.0156 (7)	0.0095 (8)	0.0088 (8)
F20	0.0267 (8)	0.0258 (8)	0.0528 (10)	0.0011 (6)	0.0146 (7)	−0.0007 (7)
C47	0.138 (10)	0.139 (9)	0.151 (9)	−0.007 (9)	0.021 (10)	0.007 (9)
Cl1	0.101 (5)	0.140 (6)	0.162 (8)	−0.006 (5)	−0.030 (5)	0.009 (5)
Cl2	0.108 (6)	0.111 (5)	0.203 (9)	−0.013 (5)	0.051 (6)	−0.024 (5)
C48	0.131 (10)	0.137 (10)	0.135 (10)	−0.007 (10)	0.027 (10)	0.003 (9)
Cl3	0.160 (9)	0.138 (7)	0.074 (5)	0.005 (6)	0.038 (5)	0.033 (5)
Cl4	0.154 (9)	0.190 (9)	0.205 (10)	−0.002 (9)	0.004 (10)	−0.027 (10)

### Geometric parameters (Å, º)

**Table:**

C1—N1	1.354 (3)	C26—F5	1.339 (3)
C1—C20	1.398 (3)	C27—C32	1.380 (4)
C1—C2	1.544 (3)	C27—C28	1.389 (4)
C2—O1	1.395 (3)	C28—F6	1.338 (3)
C2—O2	1.416 (3)	C28—C29	1.381 (3)
C2—H2	1.0000	C29—F7	1.339 (3)
C3—O3	1.394 (3)	C29—C30	1.379 (4)
C3—O2	1.404 (3)	C30—F8	1.338 (3)
C3—C4	1.523 (3)	C30—C31	1.370 (4)
C3—H3	1.0000	C31—F9	1.343 (3)
C4—N1	1.352 (3)	C31—C32	1.383 (4)
C4—C5	1.395 (3)	C32—F10	1.347 (3)
C5—C6	1.408 (3)	C33—C38	1.382 (4)
C5—C21	1.505 (3)	C33—C34	1.388 (3)
C6—N2	1.368 (3)	C34—F11	1.334 (3)
C6—C7	1.429 (3)	C34—C35	1.382 (4)
C7—C8	1.359 (4)	C35—F12	1.341 (3)
C7—H7	0.9500	C35—C36	1.369 (4)
C8—C9	1.425 (3)	C36—F13	1.338 (3)
C8—H8	0.9500	C36—C37	1.376 (4)
C9—N2	1.380 (3)	C37—F14	1.339 (3)
C9—C10	1.393 (3)	C37—C38	1.385 (4)
C10—C11	1.401 (3)	C38—F15	1.342 (3)
C10—C27	1.496 (3)	C39—C44	1.383 (3)
C11—N3	1.361 (3)	C39—C40	1.385 (3)
C11—C12	1.447 (3)	C40—F16	1.337 (3)
C12—C13	1.348 (4)	C40—C41	1.381 (4)
C12—H12	0.9500	C41—F17	1.345 (3)
C13—C14	1.449 (3)	C41—C42	1.372 (4)
C13—H13	0.9500	C42—F18	1.344 (3)
C14—N3	1.366 (3)	C42—C43	1.376 (4)
C14—C15	1.409 (3)	C43—F19	1.338 (3)
C15—C16	1.384 (3)	C43—C44	1.381 (3)
C15—C33	1.501 (3)	C44—F20	1.343 (3)
C16—N4	1.374 (3)	C45—O1	1.432 (3)
C16—C17	1.421 (3)	C45—H45A	0.9800
C17—C18	1.357 (4)	C45—H45B	0.9800
C17—H17	0.9500	C45—H45C	0.9800
C18—C19	1.429 (3)	C46—O3	1.434 (3)
C18—H18	0.9500	C46—H46A	0.9800
C19—N4	1.368 (3)	C46—H46B	0.9800
C19—C20	1.406 (3)	C46—H46C	0.9800
C20—C39	1.505 (3)	N2—H2A	0.85 (3)
C21—C26	1.380 (4)	N4—H4	0.79 (3)
C21—C22	1.386 (4)	C47—Cl1	1.781 (19)
C22—F1	1.336 (3)	C47—Cl2	1.80 (2)
C22—C23	1.383 (4)	C47—H47A	0.9900
C23—F2	1.340 (3)	C47—H47B	0.9900
C23—C24	1.376 (4)	C48—Cl3	1.705 (19)
C24—F3	1.333 (3)	C48—Cl4	1.708 (18)
C24—C25	1.373 (4)	C48—H48A	0.9900
C25—F4	1.338 (4)	C48—H48B	0.9900
C25—C26	1.393 (4)		
			
N1—C1—C20	123.2 (2)	F6—C28—C29	118.1 (2)
N1—C1—C2	122.3 (2)	F6—C28—C27	119.7 (2)
C20—C1—C2	114.6 (2)	C29—C28—C27	122.3 (2)
O1—C2—O2	107.04 (19)	F7—C29—C30	120.1 (2)
O1—C2—C1	112.86 (19)	F7—C29—C28	120.4 (2)
O2—C2—C1	113.33 (19)	C30—C29—C28	119.5 (2)
O1—C2—H2	107.8	F8—C30—C31	119.8 (2)
O2—C2—H2	107.8	F8—C30—C29	120.3 (2)
C1—C2—H2	107.8	C31—C30—C29	119.9 (2)
O3—C3—O2	113.30 (19)	F9—C31—C30	119.8 (2)
O3—C3—C4	107.60 (19)	F9—C31—C32	120.8 (2)
O2—C3—C4	109.84 (19)	C30—C31—C32	119.4 (2)
O3—C3—H3	108.7	F10—C32—C27	119.8 (2)
O2—C3—H3	108.7	F10—C32—C31	117.5 (2)
C4—C3—H3	108.7	C27—C32—C31	122.7 (2)
N1—C4—C5	124.7 (2)	C38—C33—C34	116.4 (2)
N1—C4—C3	118.3 (2)	C38—C33—C15	122.0 (2)
C5—C4—C3	117.0 (2)	C34—C33—C15	121.6 (2)
C4—C5—C6	128.7 (2)	F11—C34—C35	117.4 (2)
C4—C5—C21	118.8 (2)	F11—C34—C33	119.9 (2)
C6—C5—C21	112.5 (2)	C35—C34—C33	122.7 (2)
N2—C6—C5	127.7 (2)	F12—C35—C36	120.3 (2)
N2—C6—C7	106.6 (2)	F12—C35—C34	120.3 (2)
C5—C6—C7	125.6 (2)	C36—C35—C34	119.4 (2)
C8—C7—C6	108.6 (2)	F13—C36—C35	120.1 (2)
C8—C7—H7	125.7	F13—C36—C37	120.3 (2)
C6—C7—H7	125.7	C35—C36—C37	119.7 (2)
C7—C8—C9	107.8 (2)	F14—C37—C36	119.6 (2)
C7—C8—H8	126.1	F14—C37—C38	120.3 (2)
C9—C8—H8	126.1	C36—C37—C38	120.2 (2)
N2—C9—C10	125.0 (2)	F15—C38—C33	119.9 (2)
N2—C9—C8	106.8 (2)	F15—C38—C37	118.3 (2)
C10—C9—C8	128.1 (2)	C33—C38—C37	121.7 (2)
C9—C10—C11	125.5 (2)	C44—C39—C40	117.0 (2)
C9—C10—C27	117.0 (2)	C44—C39—C20	120.9 (2)
C11—C10—C27	117.5 (2)	C40—C39—C20	122.1 (2)
N3—C11—C10	125.1 (2)	F16—C40—C41	118.6 (2)
N3—C11—C12	111.2 (2)	F16—C40—C39	119.5 (2)
C10—C11—C12	123.4 (2)	C41—C40—C39	121.9 (2)
C13—C12—C11	106.5 (2)	F17—C41—C42	119.9 (2)
C13—C12—H12	126.7	F17—C41—C40	120.7 (2)
C11—C12—H12	126.7	C42—C41—C40	119.4 (2)
C12—C13—C14	106.1 (2)	F18—C42—C41	119.8 (2)
C12—C13—H13	127.0	F18—C42—C43	119.7 (2)
C14—C13—H13	127.0	C41—C42—C43	120.5 (2)
N3—C14—C15	124.1 (2)	F19—C43—C42	119.8 (2)
N3—C14—C13	111.3 (2)	F19—C43—C44	121.2 (2)
C15—C14—C13	124.5 (2)	C42—C43—C44	119.0 (2)
C16—C15—C14	125.3 (2)	F20—C44—C43	117.8 (2)
C16—C15—C33	117.3 (2)	F20—C44—C39	119.9 (2)
C14—C15—C33	117.2 (2)	C43—C44—C39	122.3 (2)
N4—C16—C15	126.0 (2)	O1—C45—H45A	109.5
N4—C16—C17	106.3 (2)	O1—C45—H45B	109.5
C15—C16—C17	127.3 (2)	H45A—C45—H45B	109.5
C18—C17—C16	108.3 (2)	O1—C45—H45C	109.5
C18—C17—H17	125.9	H45A—C45—H45C	109.5
C16—C17—H17	125.9	H45B—C45—H45C	109.5
C17—C18—C19	108.6 (2)	O3—C46—H46A	109.5
C17—C18—H18	125.7	O3—C46—H46B	109.5
C19—C18—H18	125.7	H46A—C46—H46B	109.5
N4—C19—C20	128.5 (2)	O3—C46—H46C	109.5
N4—C19—C18	105.8 (2)	H46A—C46—H46C	109.5
C20—C19—C18	125.7 (2)	H46B—C46—H46C	109.5
C1—C20—C19	129.1 (2)	C4—N1—C1	116.4 (2)
C1—C20—C39	118.4 (2)	C6—N2—C9	110.1 (2)
C19—C20—C39	112.42 (19)	C6—N2—H2A	123 (2)
C26—C21—C22	116.4 (2)	C9—N2—H2A	126 (2)
C26—C21—C5	121.1 (2)	C11—N3—C14	104.84 (19)
C22—C21—C5	122.5 (2)	C19—N4—C16	110.9 (2)
F1—C22—C23	118.3 (3)	C19—N4—H4	125 (2)
F1—C22—C21	119.4 (2)	C16—N4—H4	124 (2)
C23—C22—C21	122.3 (3)	C2—O1—C45	114.4 (2)
F2—C23—C24	120.5 (2)	C3—O2—C2	114.19 (18)
F2—C23—C22	120.0 (3)	C3—O3—C46	111.6 (2)
C24—C23—C22	119.5 (3)	Cl1—C47—Cl2	101.8 (12)
F3—C24—C25	120.0 (3)	Cl1—C47—H47A	111.4
F3—C24—C23	119.8 (3)	Cl2—C47—H47A	111.4
C25—C24—C23	120.2 (2)	Cl1—C47—H47B	111.4
F4—C25—C24	120.9 (2)	Cl2—C47—H47B	111.4
F4—C25—C26	120.1 (3)	H47A—C47—H47B	109.3
C24—C25—C26	119.0 (3)	Cl3—C48—Cl4	130 (2)
F5—C26—C21	120.2 (2)	Cl3—C48—H48A	104.7
F5—C26—C25	117.2 (3)	Cl4—C48—H48A	104.7
C21—C26—C25	122.6 (3)	Cl3—C48—H48B	104.7
C32—C27—C28	116.2 (2)	Cl4—C48—H48B	104.7
C32—C27—C10	121.8 (2)	H48A—C48—H48B	105.7
C28—C27—C10	121.9 (2)		
			
N1—C1—C2—O1	−111.2 (2)	C28—C29—C30—F8	−178.8 (2)
C20—C1—C2—O1	67.6 (3)	F7—C29—C30—C31	179.0 (2)
N1—C1—C2—O2	10.7 (3)	C28—C29—C30—C31	−0.3 (4)
C20—C1—C2—O2	−170.5 (2)	F8—C30—C31—F9	−1.3 (4)
O3—C3—C4—N1	−74.8 (3)	C29—C30—C31—F9	−179.8 (2)
O2—C3—C4—N1	49.0 (3)	F8—C30—C31—C32	178.0 (2)
O3—C3—C4—C5	104.3 (2)	C29—C30—C31—C32	−0.5 (4)
O2—C3—C4—C5	−131.9 (2)	C28—C27—C32—F10	−179.4 (2)
N1—C4—C5—C6	−4.2 (4)	C10—C27—C32—F10	−1.8 (4)
C3—C4—C5—C6	176.8 (3)	C28—C27—C32—C31	0.9 (4)
N1—C4—C5—C21	177.9 (2)	C10—C27—C32—C31	178.5 (3)
C3—C4—C5—C21	−1.1 (3)	F9—C31—C32—F10	−0.3 (4)
C4—C5—C6—N2	12.1 (5)	C30—C31—C32—F10	−179.5 (2)
C21—C5—C6—N2	−169.9 (3)	F9—C31—C32—C27	179.4 (3)
C4—C5—C6—C7	−165.1 (3)	C30—C31—C32—C27	0.2 (4)
C21—C5—C6—C7	12.9 (4)	C16—C15—C33—C38	74.6 (3)
N2—C6—C7—C8	−1.1 (3)	C14—C15—C33—C38	−109.9 (3)
C5—C6—C7—C8	176.6 (3)	C16—C15—C33—C34	−105.7 (3)
C6—C7—C8—C9	2.1 (3)	C14—C15—C33—C34	69.8 (3)
C7—C8—C9—N2	−2.3 (3)	C38—C33—C34—F11	−179.0 (2)
C7—C8—C9—C10	174.6 (3)	C15—C33—C34—F11	1.2 (4)
N2—C9—C10—C11	5.7 (4)	C38—C33—C34—C35	1.7 (4)
C8—C9—C10—C11	−170.7 (3)	C15—C33—C34—C35	−178.1 (2)
N2—C9—C10—C27	−178.0 (2)	F11—C34—C35—F12	0.1 (4)
C8—C9—C10—C27	5.6 (4)	C33—C34—C35—F12	179.4 (2)
C9—C10—C11—N3	−4.9 (4)	F11—C34—C35—C36	−179.5 (3)
C27—C10—C11—N3	178.7 (2)	C33—C34—C35—C36	−0.1 (4)
C9—C10—C11—C12	168.7 (3)	F12—C35—C36—F13	−1.0 (4)
C27—C10—C11—C12	−7.6 (4)	C34—C35—C36—F13	178.6 (3)
N3—C11—C12—C13	2.2 (3)	F12—C35—C36—C37	179.3 (3)
C10—C11—C12—C13	−172.2 (2)	C34—C35—C36—C37	−1.1 (4)
C11—C12—C13—C14	−2.1 (3)	F13—C36—C37—F14	1.0 (4)
C12—C13—C14—N3	1.5 (3)	C35—C36—C37—F14	−179.3 (3)
C12—C13—C14—C15	−175.6 (2)	F13—C36—C37—C38	−178.9 (3)
N3—C14—C15—C16	−5.2 (4)	C35—C36—C37—C38	0.8 (5)
C13—C14—C15—C16	171.5 (2)	C34—C33—C38—F15	179.0 (2)
N3—C14—C15—C33	179.7 (2)	C15—C33—C38—F15	−1.3 (4)
C13—C14—C15—C33	−3.5 (4)	C34—C33—C38—C37	−2.0 (4)
C14—C15—C16—N4	2.9 (4)	C15—C33—C38—C37	177.8 (3)
C33—C15—C16—N4	177.9 (2)	F14—C37—C38—F15	0.0 (4)
C14—C15—C16—C17	−169.0 (3)	C36—C37—C38—F15	179.8 (3)
C33—C15—C16—C17	6.1 (4)	F14—C37—C38—C33	−179.1 (3)
N4—C16—C17—C18	−2.1 (3)	C36—C37—C38—C33	0.8 (4)
C15—C16—C17—C18	171.0 (3)	C1—C20—C39—C44	−94.8 (3)
C16—C17—C18—C19	0.9 (3)	C19—C20—C39—C44	87.5 (3)
C17—C18—C19—N4	0.6 (3)	C1—C20—C39—C40	88.2 (3)
C17—C18—C19—C20	−179.1 (2)	C19—C20—C39—C40	−89.5 (3)
N1—C1—C20—C19	−3.4 (4)	C44—C39—C40—F16	179.6 (2)
C2—C1—C20—C19	177.8 (2)	C20—C39—C40—F16	−3.2 (4)
N1—C1—C20—C39	179.3 (2)	C44—C39—C40—C41	0.3 (4)
C2—C1—C20—C39	0.5 (3)	C20—C39—C40—C41	177.4 (2)
N4—C19—C20—C1	12.6 (4)	F16—C40—C41—F17	0.9 (4)
C18—C19—C20—C1	−167.7 (3)	C39—C40—C41—F17	−179.8 (2)
N4—C19—C20—C39	−170.0 (2)	F16—C40—C41—C42	−179.8 (2)
C18—C19—C20—C39	9.7 (3)	C39—C40—C41—C42	−0.5 (4)
C4—C5—C21—C26	−95.9 (3)	F17—C41—C42—F18	−0.2 (4)
C6—C5—C21—C26	85.8 (3)	C40—C41—C42—F18	−179.5 (2)
C4—C5—C21—C22	87.8 (3)	F17—C41—C42—C43	179.7 (2)
C6—C5—C21—C22	−90.4 (3)	C40—C41—C42—C43	0.4 (4)
C26—C21—C22—F1	−179.3 (2)	F18—C42—C43—F19	−1.2 (4)
C5—C21—C22—F1	−2.9 (4)	C41—C42—C43—F19	178.9 (2)
C26—C21—C22—C23	0.3 (4)	F18—C42—C43—C44	179.7 (2)
C5—C21—C22—C23	176.7 (2)	C41—C42—C43—C44	−0.2 (4)
F1—C22—C23—F2	−0.8 (4)	F19—C43—C44—F20	0.8 (4)
C21—C22—C23—F2	179.6 (2)	C42—C43—C44—F20	179.9 (2)
F1—C22—C23—C24	178.8 (3)	F19—C43—C44—C39	−179.1 (2)
C21—C22—C23—C24	−0.8 (4)	C42—C43—C44—C39	0.0 (4)
F2—C23—C24—F3	1.4 (4)	C40—C39—C44—F20	−179.9 (2)
C22—C23—C24—F3	−178.3 (3)	C20—C39—C44—F20	2.9 (3)
F2—C23—C24—C25	179.5 (3)	C40—C39—C44—C43	0.0 (4)
C22—C23—C24—C25	−0.1 (4)	C20—C39—C44—C43	−177.2 (2)
F3—C24—C25—F4	−0.2 (5)	C5—C4—N1—C1	169.9 (2)
C23—C24—C25—F4	−178.4 (3)	C3—C4—N1—C1	−11.1 (3)
F3—C24—C25—C26	179.5 (3)	C20—C1—N1—C4	162.6 (2)
C23—C24—C25—C26	1.3 (5)	C2—C1—N1—C4	−18.7 (3)
C22—C21—C26—F5	178.6 (2)	C5—C6—N2—C9	−178.0 (3)
C5—C21—C26—F5	2.2 (4)	C7—C6—N2—C9	−0.4 (3)
C22—C21—C26—C25	1.0 (4)	C10—C9—N2—C6	−175.4 (3)
C5—C21—C26—C25	−175.5 (3)	C8—C9—N2—C6	1.6 (3)
F4—C25—C26—F5	0.2 (4)	C10—C11—N3—C14	173.1 (2)
C24—C25—C26—F5	−179.5 (3)	C12—C11—N3—C14	−1.2 (3)
F4—C25—C26—C21	177.9 (3)	C15—C14—N3—C11	177.0 (2)
C24—C25—C26—C21	−1.8 (5)	C13—C14—N3—C11	−0.1 (3)
C9—C10—C27—C32	113.0 (3)	C20—C19—N4—C16	177.7 (2)
C11—C10—C27—C32	−70.3 (3)	C18—C19—N4—C16	−2.1 (3)
C9—C10—C27—C28	−69.5 (3)	C15—C16—N4—C19	−170.6 (2)
C11—C10—C27—C28	107.1 (3)	C17—C16—N4—C19	2.6 (3)
C32—C27—C28—F6	179.1 (2)	O2—C2—O1—C45	−66.4 (2)
C10—C27—C28—F6	1.5 (4)	C1—C2—O1—C45	59.0 (3)
C32—C27—C28—C29	−1.8 (4)	O3—C3—O2—C2	64.5 (2)
C10—C27—C28—C29	−179.4 (2)	C4—C3—O2—C2	−55.9 (3)
F6—C28—C29—F7	1.4 (4)	O1—C2—O2—C3	153.83 (19)
C27—C28—C29—F7	−177.8 (2)	C1—C2—O2—C3	28.8 (3)
F6—C28—C29—C30	−179.3 (2)	O2—C3—O3—C46	67.4 (3)
C27—C28—C29—C30	1.5 (4)	C4—C3—O3—C46	−171.0 (2)
F7—C29—C30—F8	0.5 (4)		

### Hydrogen-bond geometry (Å, º)

**Table:**

*D*—H···*A*	*D*—H	H···*A*	*D*···*A*	*D*—H···*A*
C8—H8···F18^i^	0.95	2.59	3.424 (3)	147
C18—H18···F8^ii^	0.95	2.51	3.319 (3)	143
C45—H45*A*···F12^iii^	0.98	2.55	3.519 (3)	169
C45—H45*B*···F19^iv^	0.98	2.66	3.389 (3)	132
C45—H45*A*···F20	0.98	2.82	3.184 (3)	103
C46—H46*A*···F10^v^	0.98	2.63	3.496 (4)	148
C46—H46*C*···F1^vi^	0.98	2.60	3.505 (4)	153
C48—H48*B*···F12^iii^	0.99	2.42	3.25 (5)	141
N2—H2*A*···N1	0.85 (3)	2.56 (3)	3.040 (3)	117 (3)
N2—H2*A*···N3	0.85 (3)	2.33 (3)	2.858 (3)	121 (3)
N4—H4···N1	0.79 (3)	2.61 (3)	3.040 (3)	116 (2)
N4—H4···N3	0.79 (3)	2.33 (3)	2.852 (3)	125 (3)

Symmetry codes: (i) *x*, *y*+1, *z*; (ii) *x*, *y*−1, *z*; (iii) −*x*+1, *y*, −*z*+1/2; (iv) −*x*+1, −*y*+1, −*z*+1; (v) *x*−1/2, *y*−1/2, *z*; (vi) −*x*+1/2, −*y*+3/2, −*z*+1.
